# Ether-Type Moieties in the Lipid Part of Glycoinositolphospholipids of *Acanthamoeba rhysodes*

**DOI:** 10.1007/s11745-014-3884-9

**Published:** 2014-02-18

**Authors:** Magdalena A. Karaś, Ryszard Russa

**Affiliations:** Department of Genetics and Microbiology, Maria Curie-Skłodowska University, Akademicka 19, 20-033 Lublin, Poland

**Keywords:** Ether lipids, Glycoinositolphospholipids, Lipophosphonoglycan, *Acanthamoeba rhysodes*, Isopropylidene derivatives, Hydrofluoric acid, Nitrous acid deamination, GC–MS, TLC, MALDI-TOF

## Abstract

Ether lipids were identified among components liberated with HF and nitrous acid deamination from *Acanthamoeba rhysodes* whole cells and its membrane glycoinositolphospholipids (GIPL). Liberated ether glycerols were converted to various derivatives that served characterization thereof. These included TMS and isopropylidene derivatives, oxidation with sodium periodate to aldehyde followed by reduction with NaBH_4_ to alcohol, and reaction of the alcohol with acetic anhydrite to form acetate derivatives. Periodate sensitivity demonstrated that the alkyl side chains were linked to the *sn*-1 position of glycerol. Combined information from TLC, GC–MS analysis, MALDI-TOF spectrometry, and chemical degradation experiments indicated the presence of ether-linked saturated normal and branched hydrocarbons with a length of C_20–23_ in the phospholipid fraction, C_20–24_ in free GPI, and C_21–23_ in the LPG polymer. The distribution of particular classes of alkylglycerols was similar for phospholipid and GPI fractions, and amounted to 2.62 % (±0.04–0.28) 1-*O*-eicosanyl-*sn*-glycerol, 16.66 % (±0.32–1.1) 1-*O*-uncosanyl-*sn*-glycerol, 9.18 % (±0.33–1.37) *anteiso*-1-*O*-docosanyl-*sn*-glycerol, 47.56 % (±0.32–2.14) 1-*O*-docosanyl-*sn*-glycerol, 20.56 % (±0.58–1.67) *anteiso*-1-*O*-tricosanyl-*sn*-glycerol, and 2.34 % (±0.12–0.63) 1-*O*-tricosanyl-*sn*-glycerol. For LPG preparation, the most abundant were *anteiso*-1-*O*-tricosanyl-*sn*-glycerol (57.26 %) and 1-*O*-docosanyl-*sn*-glycerol (30.12 %). The data from TLC and GC–MS analysis showed that ether lipids from phospholipids probably represent the *lyso*-alkylglycerol type, while those derived from GIPL are alkylacylglycerol moieties.

## Introduction

1-*O*-Alkyl-*sn*-glycerols usually form the backbone of complex ether-linked glycerolipids, including biologically active lipids e.g. PAF (platelet-activating factor) and plasmalogens [1]. They were also identified in parasite *Leishmania* lipophosphoglycan and glycoinositol phospholipids as 1-alkyl-2-acylglycerol and *lyso*-alkyl phosphatidylinositol backbones. These ether-type lipid moieties were unusual since they contained predominantly 18:0, 22:0, 24:0, or 26:0 alkyl chains. An increase in the content of 24:0 and 26:0 alkyls was observed with elongation of the carbohydrate chain and appeared almost exclusively in lipophosphoglycan [2, 3]. The existence of long alkyl chains in ether lipids is a rare feature. In general, alkyl chains contain an even number of carbons ranging from C14 to C22 (both saturated or monounsaturated), and saturated or monounsaturated *O*-alkyl chains of 16 and 18 carbon atoms are the most prevalent. Odd-numbered polyunsaturated and branched chains are only minor components [1].

It has been demonstrated that synthetic alkylacylglycerols corresponding to lipid parts of *Leishmania donovani* GPI [4], those derived from products of *Leishmania* lipophosphoglycan hydrolysis as 1-*O*-alkyl-2-*lyso*-glycerol [5] as well as the major surface molecules (glycoinositolphospholipids and lipophosphoglycan) isolated from the parasite cells and bearing ether lipids, are antagonists of protein kinase C (PKC) in vitro [4, 5]. Taken together, it was proposed that ether lipid moieties and not the carbohydrate domain could modulate the signalling pathways. Short-chain alkylglycerols have also been shown to increase permeability of tight junctions in the blood–brain barrier (BBB) [6] and facilitate transport of some drugs [7]. Successful treatment of many brain disorders seems to be impossible because of very limited penetrations of drugs across the BBB, but the demonstrated property of alkylglycerols suggests that they possess several potent pharmacological activities.

Glycoinositolphospholipids (GIPL) are heteropolymers in which the sugar portion is coupled to the lipid moiety via an intermediate inositol phosphate. In turn, glycosylphosphatidylinositol (GPI) anchors are a class of GIPL which carry a conservative core structure “Manα1-2Manα1-6Manα1-4GlcNH_2_α1-6*myo*-inositol-1-PO_4_-lipid”. The lipid moiety in the GPI structure can vary in the nature and the core glycan can have side-chain modifications such as ethanolamine phosphate, mannose, galactose, or sialic acid [8]. The chemical composition of the aliphatic residues is dependent on the organism and the stage in its life cycle. In a majority of eukaryotes, GPI are responsible for binding with a wide group of plasma membrane proteins. In protozoan organisms, they exist in the membrane as free or carry a carbohydrate moiety, e.g. in the lipophosphoglycan of *Leishmania* and lipophosphonoglycan (LPG) of *Acanthamoeba.* In *Leishmania* they are based on a type-2 GPI core, Manα1-3Manα1-4GlcNH_2_α1-6 PtdIns [9].

*Acanthamoeba* spp. belongs to parasitic protozoa responsible mainly for granulomatous amoebic encephalitis (GAE) and acanthamoeba keratitis (AK). The lipid content and isolation, purification, and partial characterization of LPG from plasma membranes of *Acanthamoeba* species have been described previously. Basic chemical analyses have contributed to establishing the composition of 77 % of LPG by weight containing: 26 % neutral sugars (Glc, Man, Gal, Xyl), 3.3 % amino sugars (GalN, GlcN), 8 % inositol, 10 % aminophosphonates (AEP, 1-OH AEP), 3.2 % acid-hydrolyzable phosphate, 14 % fatty acids, and 13 % long chain fatty acids [10–12]. In those reports, the described lipid backbone of LPG was the ceramide-type. In the present study, we pre-analyzed lipids liberated with HF from whole cells of *Acanthamoeba rhysodes* but focused on these derived from isolated GIPL (LPG and free GPI). Among lipids released from whole cells, we identified *lyso*-alkylglycerols with saturated normal and branched hydrocarbons with a length of C_20–23_ as the side chain, which probably came from phospholipids. In the GPI fraction, alkyls with a length of C_21–24_ occurred, and the distribution of the particular classes was similar to that obtained from phospholipids. In contrast, only C_21–23_ AKG were identified in the LPG preparation. The most abundant among the ether lipids discovered in *A. rhysodes* were 1-*O*-docosanyl-*sn*-glycerol in phospholipid and GPI fractions and *anteiso*-1-*O*-tricosanyl-*sn*-glycerol in LPG, respectively. To our knowledge, this is the first report of this kind of ether lipids in *Acanthamoeba*.

## Materials and Methods

### Microorganisms and Culture Conditions

*Acanthamoeba rhysodes* (*Hartmannella rhysodes*) Chang-strain was obtained from the culture collection of Poznań University, Poland. The endocytobiont free amoebae were grown axenically in 300-mL Erlenmeyer flasks containing 100 mL of PYG (peptone yeast glucose) medium, pH 6.6. The chemical composition of the PYG medium was essentially the same as that described by Band [13] and consisted of 15 g proteose peptone 3 (Difco), 5 g yeast extract (Difco), 10 g glucose, 120 mg NaCl, 3 mg MgCl_2_ 6H_2_O, 3 mg CaCl_2_, 3 mg FeSO_4_, 142 mg Na_2_HPO_4_, and 136 mg KH_2_PO_4_ in 1 L. The culture was incubated on a rotary shaker with an acentric rotation of 3 cm (120 rev/min) at 28 °C. Amoebae from the early stationary phase of growth were harvested by repeated centrifugation at 300×*g* for 10 min with washing 0.15 M KCl. Samples of whole cells submitted to hydrolysis (4 M HCl/100 °C/4 h) and *n*-hexane soluble products as TMS derivatives were analyzed in GC–MS.

### Extraction and Purification of Glycoinositolphospholipids

**Scheme 1:**
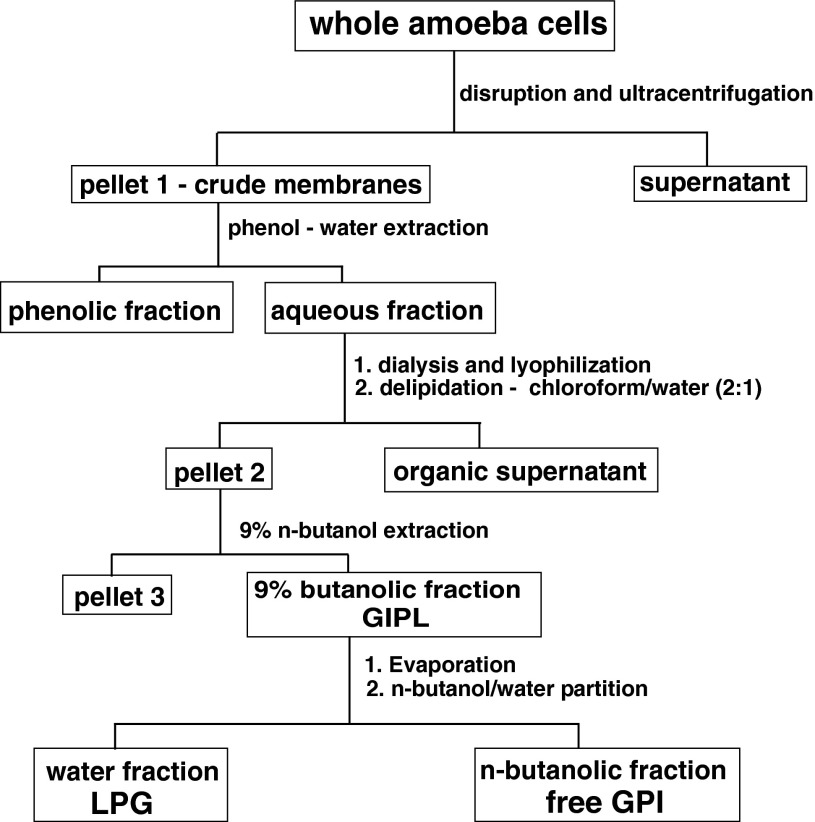
Obtaining GIPL, LPG, and GPI fractions from whole amoeba cells

Glycoinositolphospholipids were purified as described earlier [14]. The saline-washed cells were suspended in two volumes of fresh 0.15 M KCl, and disrupted in a Potter disintegrator in ice. The homogenate was ultracentrifuged for 3 h at 123,500*g* (Beckman rotor Type 50.2 Ti) [11]. The sediment (a crude membrane pellet) was treated with phenol—water [15] at 65 °C three times. The combined aqueous phases of the hot phenol–water extraction were dialyzed against tap water and lyophilized. The deposited material was then extracted with 20 volumes of mixture chloroform/MeOH (2:1, by vol.) according to the method of Korn et al. [11] to remove traces of phospholipids, washed with acetone, washed twice with water, and lyophilized. The delipidated material was extracted three times with 9 % *n*-butanol for 3 h, centrifuged, and the supernatants obtained were combined and rotary evaporated. Then, the GIPL obtained were subjected to *n*-butanol/water (1:1, by vol.) partition [16]. The water phase was washed twice with a fresh portion of butanol. During that process, LPG (the aqueous phase) was separated from residual free GPI (butanol phase) (Scheme 1). Both phases were dried and analyzed. The efficiency of the purification protocol for LPG was assessed by SDS—polyacrylamide gel electrophoresis and GC–MS analysis of fatty acids [14].

### Releasing the Glycerol Lipids with HF

Briefly, (1) 30 mg of dry cells, (2) 20 mg of GIPL were treated with 1 mL (250 μL for GIPL) of ice-cold HF for 48 h at 0–4 °C with mixing. The hydrofluoric acid was removed by nitrogen flushing. The pellets obtained were subjected to extractions: chloroform/water (1:1 by vol.; 1 mL each) for lipids liberated from whole amoeba cells, and with subsequent organic solvents: chloroform, hexane, diethyl ether (1 mL each), followed by partition with the water/butanol system 1:1 by vol.; 1 mL each for lipids liberated from GIPL. Organic phases for particular samples were combined and dried with a flush of nitrogen and subjected for further analyses.

### Characterization of Ether Lipids Liberated with HF

Conversion of small portions of lipids liberated with HF from whole cells and separated in TLC using the solvent system chloroform/MeOH (1:1, by vol., solvent 1) [12] was conducted to obtain (A) TMS and (B) isopropylidene derivatives [17, 18]. To obtain the isopropylidene derivative, the sample of lipids was dissolved in 1 mL of acetone, followed by addition of 5 μL of concentrated HClO_4_, and incubated at room temperature for 15 min. Then, 1 M ammonium hydroxide (40 μL) was added for neutralization. The sample was brought to dryness, which was followed by partitioning with 2 mL each of chloroform and water. The chloroform-soluble products were washed twice with water, dried over sodium sulfate, and concentrated under a flux of nitrogen. The authentic standards (both 25 μg): butyl alcohol (Aldrich) and *N*-tetracosanoyl-phytosphingosine (Matreya) were also converted to an isopropylidene derivative. As isopropylidene derivatives, the sample and standards were subjected to TLC in the solvent system hexane/diethyl ether/acetic acid, 80:20:1, by vol., (solvent 2) [19], and GC–MS (except the ceramide derivative). Additionally, the TMS derivatives were analyzed by GC–MS.

The samples of HF-released lipids were subjected to solvolysis with 3 M HCl in MeOH for 18 h at 80 °C and 1 M KOH in MeOH for 18 h at 100 °C [17]. The products were partitioned with chloroform/water in a final ratio of 1:1. Organic solvent extracts were dried under a stream of nitrogen and the lipophilic products obtained were converted to TMS derivatives, which was followed by GC–MS analyses.

For ether cleavage, the sample of ether lipids liberated with HF was treated with HI according to the Orlandi and Turco [17] method. An aliquot was dissolved in 1 mL of hydroiodic acid (57 %) and treated for 18 h at 120 °C under a nitrogen atmosphere. Then, it was extracted three times with 2 mL of diethyl ether. The combined ether extracts were washed successively with water and saturated potassium bicarbonate to remove excess hydroiodic acid. Free iodine was removed by extraction with a 50 % solution of sodium thiosulfate. The ether extract (of alkyl iodides) was cleaned in column chromatography on silica gel with *n*-hexane elution [20], then dried, dissolved in chloroform, and analyzed by GC–MS.

HF released lipids were also treated with sodium periodate followed by reduction and acetylation according to Orlandi and Turco [17]. The sample (30 μg) was dissolved in 0.5 mL of a chloroform/MeOH/water (16:16:5, by vol.) mixture adjusted to pH 9.5 with NH_4_OH, to which 0.5 mL of 0.057 M sodium periodate was added in the same solution. After incubation in the dark at room temperature for 16 h, 1 mL of MeOH/water (1:1, by vol.) and 1.5 mL of chloroform were added to the reaction mixture, vortexed, and centrifuged. The organic phase was taken to dryness under a stream of nitrogen, redissolved in 0.5 mL of MeOH, and reduced with 0.5 mL of 2.64 M NaBH_4_ in 0.04 M NaOH for 1 h at 45 °C. The reaction mixture was then partitioned after addition of 0.03 mL of 1 M HCl, 0.52 mL of H_2_O, 0.3 mL of MeOH, and 1.6 mL of chloroform. The organic solvent phase was washed twice with 0.5 mL H_2_O, taken to dryness under a stream of nitrogen, and acetylated with acetic anhydrite/pyridine (1:1, by vol.) for 0.5 h at 100 °C. After suspension in *n*-hexane, the alkylglycerol acetates obtained were analyzed by GC–MS. The same treatment was applied to 25 μg of the authentic standard of butyl alcohol (Aldrich).

### Analysis of Ether Lipids Liberated from of Glycoinositolphospholipids

Samples of (1) GIPL, (2) purified LPG, and (3) GIP were hydrolyzed according to the method of Watanabe et al. [21]. Briefly, 5 mg of the appropriate sample was treated with 1 mL of the MeOH/water/HCl mixture (11:2.6:1, by vol.) at 80 °C overnight, and the fatty acid methyl esters (FAME) combined with ether lipids were extracted into *n*-hexane, and derivatized with TMS reagent prior to GC–MS analysis. 25 μg of the authentic standard of butyl alcohol was converted to TMS derivatives as well and analyzed in GC–MS.

Lipids liberated from GIPL with HF subjected to: (A) conversion to isopropylidene derivatives and TLC in solvent 2, (B) acid hydrolysis followed by conversion to isopropylidene and TLC chromatography in solvent 2, (C) conversion to TMS derivatives and GC–MS analysis, (D) acid hydrolysis, hexane/water extraction, and conversion of organic soluble products to TMS derivatives and GC–MS analysis.

The sample of LPG was subjected to deamination according to Caroff et al. [22] with a minor modification. Briefly, about 25 mg of the preparation was suspended in 1.25 mL of water and equal volumes of 33 % acetic acid and 5 % sodium nitrite. After 2.5 h of incubation at 37 °C, a fresh portion (0.2 mL) of acetic acid and sodium nitrite was added, and the incubation continued for another hour. The lipids liberated during deamination were extracted with *n*-butanol (7 mL). The aqueous phase was rinsed twice with organic solvent and combined butanol fractions were dried under reduced pressure. After desalting on Dowex-50 W H^+^, the water-soluble deaminated products were freeze-dried, reduced (NaBH_4_), desalted again on a Dowex-50 W H^+^ column, and co-distilled with MeOH. Aliquots of the material were subjected to hydrolysis (2 M TFA/120 °C/2 h), reduction, and peracetylation. The butanolic fraction of lipids liberated with deamination was submitted to acid hydrolysis (4 M HCl/100 °C/4 h) followed by extraction with *n*-hexane, and conversion to TMS derivatives. The aqueous acid hydrolysate was dried and converted to an *O*-trimethylsilyl derivative with BSTFA/pyridine (1:1, by vol.) for an hour at 20–22 °C. All the samples were analyzed by GC–MS.

### Analytical Methods

TLC was performed using Silica gel 60 F_254_ (Merck) plates with the solvents 1 and 2. Chromatograms were visualized with iodine vapour for the tested samples and with 10 % H_2_SO_4_ in 70 % methanol for the authentic standards. The initial concentration of the samples and standards were 25 mg/mL. Individual lipid classes liberated with HF were separated by preparative TLC using solvent 1. The bands obtained were scraped and eluted from the silicic acid with chloroform/MeOH/water (25:15:2.5, by vol.), filtered through Whatman No. 1, suspended in chloroform, and kept in 4 °C for further analysis [12]. Aliquots from separated fractions (samples A–D) were subjected for further analyses by GC–MS. Sample D was also analyzed by MALDI-TOFMS. Isopropylidene derivatives were separated in TLC with solvent 2 and eluted from silicic acid with the same mixture prior to analysis.

The LPG preparation (5 μg) was separated in 12.5 % SDS–tricine polyacrylamide electrophoresis gel [23] and the bands were visualized by silver staining after oxidation with periodate according to the method of Tsai and Frasch [24].

MALDI-TOF was performed with the Zenker et al. [25] method using acetonitrile as a solvent to obtain a 50 % (v/v) 2,5-dihydrobenzoic acid (DHB) matrix on a Voyager-Elite (PE Biosystems) instrument fitted with a VSL-337 ND nitrogen laser (337 nm) and operated in the linear mode, at an accelerating potential of 20 kV, with positive or negative detection. The spectra obtained were the averages of 150 scans. Then, 12.5 μg of the sample (from spot D) was mixed with an equal volume of the DHB matrix solution, and spotted and dried on a MALDI-TOF sample plate.

The TMS and isopropylidene alkylglycerol derivatives, alkyl iodides, and alkylacetylglycols derived during periodate oxidation were analyzed by GC–MS in the EI mode. The analysis was performed on an Agilent 7890A-5975C instrument equipped with a capillary column (HP-5MS, 30 m × 0.25 mm), applying a temperature gradient of 150 °C (5 min) to 310 °C at 5 °C min^−1^.

## Results

### Analysis of Acid Hydrolysis Products from Whole Amoeba Cells: Ether Lipids

Among the acid hydrolysis products liberated from whole amoeba cells and extracted with hexane, ether lipids were also identified by GC–MS. The analysis of the chromatographic data indicated the presence of seven species of alkylglycerols with ether-linked saturated normal and branched hydrocarbons with a length of C_20–23_ (n20:0 4.8 %, b21:0 1 %, n21:0 23 %, b22:0 5.6 %, n22:0 42.4 %, b23:0 18.4 %, b23:0 4.8 %). Ether lipids with normal alkyl chains accounted for around 75 % while those with branched chains represented 25 %. The AKG with a branched alkyl residue were assigned to the *anteiso*-type on the basis of their retention times with comparison to the retention time of the authentic standard and published data [26].

Complete GC–MS spectra were recorded for the TMS ethers of monoalkylglycerols. The characteristic base ion for 1-*O*-alkyl-glycerol of the TMS derivatives was located at 205 *m*/*z*. The TMS ethers of saturated monoalkylglycerols gave no or very little molecular ion. However, ions for [M-15], [M-90], [M-147] were quite abundant and characteristic for this kind of derivatives (summarized in Table 1). No peak for 2-*O*-alkyl-glycerol was identified. Assignment of the monoalkylglycerols followed the MS fragmentation patterns published by Bertello et al. [27], Myher et al. [28], and Orlandi and Turco [17].

**Table 1:** Fragmentation ions obtained in the spectra of TMS ether derivatives of monoalkylglycerols in GC–MS analysis

Fragment ion (*m*/*z*)	1-Monoalkylglycerols						
	n20:0	b21:0	n21:0	b22:0	n22:0	b23:0	n23:0
M-15	501	nd	515	529	529	543	543
M-90	426	nd	440	454	454	468	468
M-104	412	426	426	440	440	454	454
M-(73 + 74)	**369**	**383**	**383**	**397**	**397**	**411**	**411**
M-(103 + 90)	323	nd	nd	nd	nd	nd	nd

The bold values represent the most characteristic ion

*n* normal chain, *b* branched chain, *nd* not detected

### Analysis of Products Liberated with HF from Whole Amoeba Cells

**Fig. 1:**
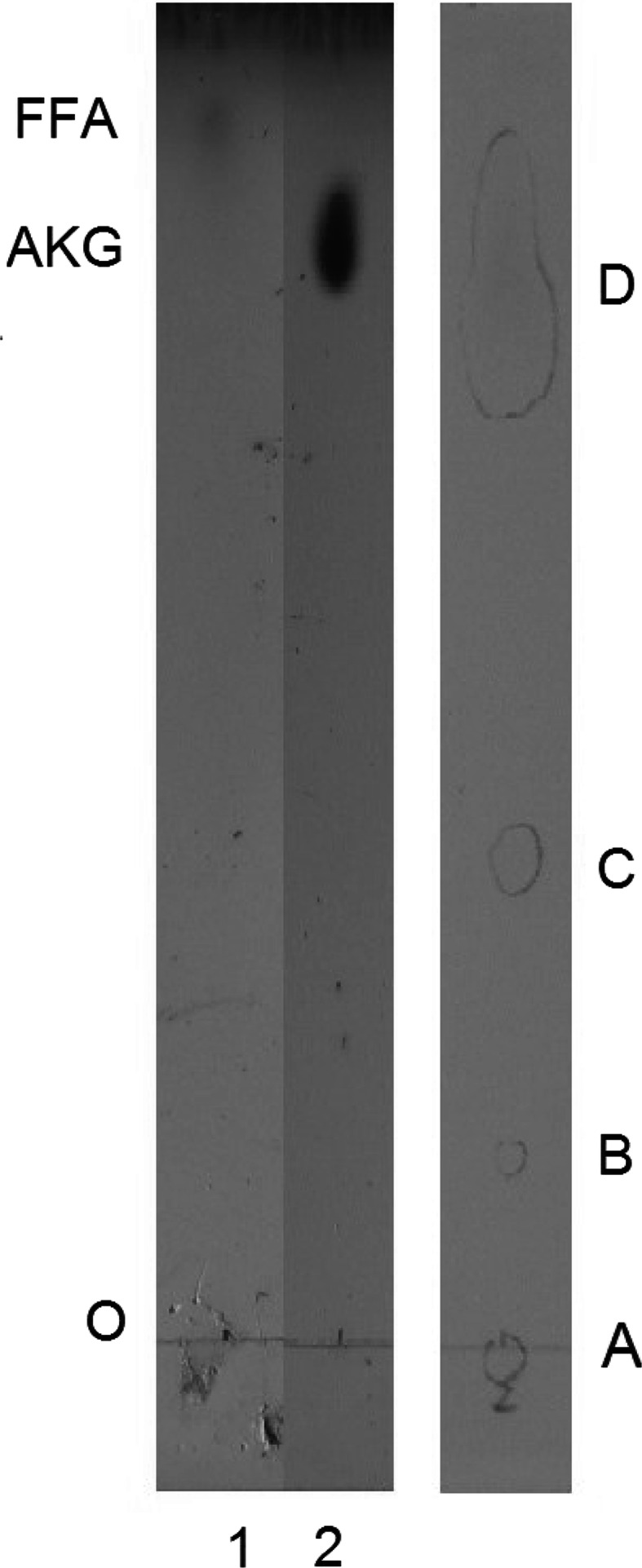
TLC analysis of lipids separated on silica gel plates (Merck) in solvent 1. *Left panel* Authentic standards visualized with sulfuric acid; *line 1* fatty acid C23:0, *line 2* AKG C18 ether glycerol. *Right panel* Lipids liberated with HF (150 μg) from *A. rhysodes* cells and visualized with iodine vapour. *FFA* Free fatty acids, *AKG* alkylglycerols, *O* origin

The residues liberated with HF from whole amoeba cells were submitted to chloroform:water extraction. Organic solvent-soluble products were separated by TLC carried out on silica gel plates, and developed with solvent 1 [12]. Four bands (A, B, C, D) with an *R*_F_ of 0.0, 0.21, 0.37, and 0.85 respectively, were detected by iodine vapor (Fig. 1). The material from appropriate bands was scraped and eluted from silicic acid, and subjected to GC–MS analysis after conversion into TMS derivatives. The ether lipids were identified only in the material derived from spot D with *R*_F_ 0.85 and their chromatographic profile was almost identical to that obtained for the material subjected to acid hydrolysis. The only minor difference was the lack of the peak for branched 1-*O*-heneicosyl-*sn*-glycerol comprising around 1 % of the whole ether lipids pool. The chromatographic analysis also indicated that, beside ether lipids, the examined material from spot D contained monoacylglycerols and free fatty acids (16:0, 18:0, 18:1), which co-migrated with them on the TLC plate. It is known that, in terms of chromatography, solubility, and certain other physical properties, glyceryl ethers behave in exactly the same way as acylglycerols [19].

The structure of monoacylglycerides was obtained by interpretation of the mass spectra of their TMS derivatives [28, 29]. In these compounds, the most abundant [M-103]^+^ fragment, formed by the heterolytic cleavage of the C_1_–C_2_ bond characteristic for 1-*sn*-monoacylglycerols [28] was considered. These were: 343, 371, 385, 399 *m*/*z* for 2,3-dihydroxypropyl myristate, palmitate, margarate, and stearate, respectively. For unsaturated fatty acids as side chains in the 1-*sn* position of the glycerol moiety, ions for [M-103]^+^ as: 369, 397, 419, 421, and 423 *m*/*z* for FA 16:1, 18:1, 20:4, 20:3, 20:2, respectively, were also identified. Four classes of 2-isomer with the characteristic ion at *m*/*z* 218 were also observed among the monoglyceride species (Table 2). They probably derived from plasmalogens in the acid environment [10]. Other characteristic fragmentation ions in the GC–MS spectra of the TMS ethers of 1- and 2-monoacylglycerols are summarized in Table 2.

**Table 2:** Fragmentation ions obtained in the spectra of TMS ether derivatives of monoacylglycerols in GC–MS analysis

Fragment ion (*m*/*z*)	1-MG									2-MG			
	14:0	16:0	17:0	18:0	16:1	18:1	20:4	20:3	20:2	14:0	16:0	18:0	18:1
M	nd	nd	nd	nd	472	500	522	524	526	nd	nd	nd	500
M-15	431	459	473	487	457	485	507	509	511	431	459	487	485
M-73	373	401	nd	429	nd	427	nd	451	nd	373	nd	429	nd
M-(73 + 74)	299	nd	nd	355	nd	353	nd	377	379	299	nd	355	nd
M-90	nd	nd	nd	nd	382	410	nd	434	436	nd	nd	nd	410
M-(71 + 90)	nd	nd	nd	nd	311	339	nd	363	365	285	313	341	339
M-103	**343**	**371**	**385**	**399**	**369**	**397**	319	421	423	nd	nd	nd	nd
M-(103 + 90)	nd	nd	nd	nd	nd	307	nd	363	365	nd	nd	nd	nd
Acyl	211	239	253	267	nd	nd	nd	nd	nd	211	239	267	265

The bold values represent the most characteristic ion

*1-MG* 1-monoacylglycerols, *2-MG* 2-monoacylglycerols, *nd* not detected

The present study confirms the prediction of Johnson and Holman [30] that unsaturated monoacylglycerols show the highest parent ion intensities. Unsaturated ethers containing double bond(s) in the chain also exhibit a more pronounced molecular ion, which was not detected for the saturated ones. Also other ions typical for TMS ethers of 1-monoacylglycerols (55, 57, 67, 69, 73, 103, 129, 147, 201, 203, 205 *m*/*z*) were identified in the spectra.

MALDI-TOFMS analysis of HF-liberated and TLC separated products (spot D, solvent 1) from whole amoeba cells confirmed the existence of ether lipids in the material. Based on the molecular ion *m*/*z* values, C_20_- and C_22_-alkylglycerols were identified in the positive mode mass spectra as ions [M + H]^+^ 373.77 and 401.01 *m*/*z*, respectively. Pseudomolecular ions [M + Na]^+^ and [M + K]^+^ for all the species of the ether lipids were also observed (for C_20_-AKG 395.03 and 411.01 *m*/*z*, C_21_-AKG 409.12 and 425.06 *m*/*z*, C_22_-AKG 423.08 and 439.03 *m*/*z*, C_23_-AKG 437.12 *m*/*z* and tr, respectively).

**Fig. 2:**
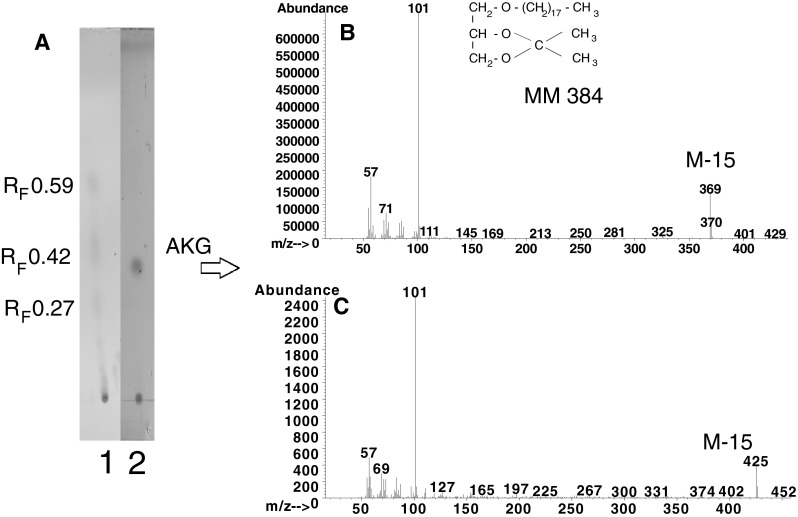
**a** TLC analysis of lipids as isopropylidene derivatives separated on silicic plates (Merck) in solvent 2. *Line 1* Lipids from spot D (Fig. 1), *line 2* Authentic standard of C18 ether glycerol. Mass spectrum of: **b** the isopropylidene derivative of the authentic standard, **c** C22 ether glycerol from *line 1*

To separate the constituents of spot D, they were converted to 1-radyl-2,3-*O*-isopropylidene derivatives. The 1-alkyl-2,3-*O*-isopropylidene derivatives were separated from 1-acyl-2,3-*O*-isopropylidene by TLC using solvent system B. Three spots with an *R*_F_ 0.27, 0.42, and 0.59 were visualized with iodine vapour (Fig. 2a). The individual spots were scraped and directly analyzed by GC–MS. The base peak in the spectra of the isopropylidene derivatives of *lyso*-glyceryl ethers at 101 *m*/*z* [31] (Fig. 2b) and the presence of ions [M-15]^+^ at EI mode of fragmentation were identified only for the compounds from the spot with *R*_F_ 0.42. The presence of the M-15 ions (loss of the methyl group) at 397, 411, 425 (Fig. 2c), and 439 *m*/*z* distinguishes the 1-*O*-alkyl moieties with eicosanoic, heneicosanoic, docosanoic and tricosanoic radyl chains, respectively. This result is in good correlation with findings obtained for TMS ether derivatives of alkylglycerols. The spot with *R*_F_ 0.27 originated from monoacylglycerols while that with *R*_F_ 0.58 from free fatty acids.

**Fig. 3:**
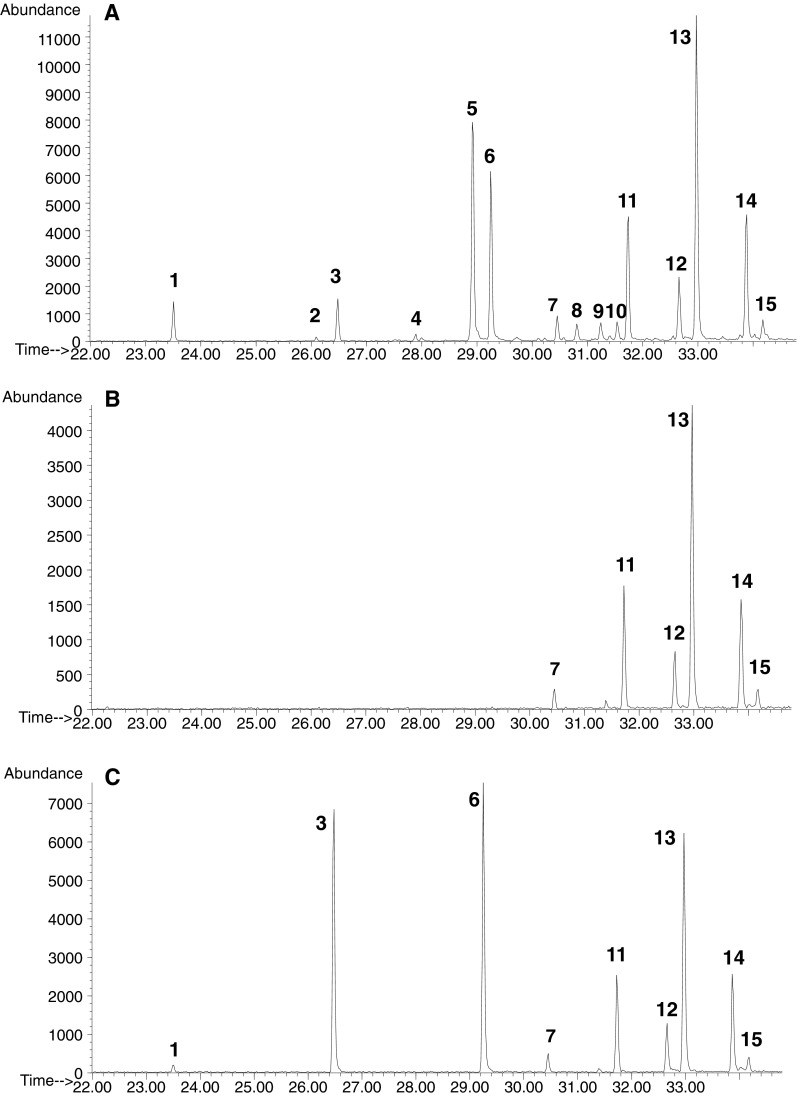
Ionograms for the ion at *m*/*z* 205 of TMS ethers of monoalkyl- and monoacylglycerols (1-*sn*) HF liberated from *A. rhysodes* cells. **a** Obtained from TLC—spot D. **b** The same sample but after acid methanolysis. **c** The same sample but after alkali methanolysis. The number of marked peaks denotes TMS derivatives *1* 2,3-dihydroxypropyl myristate; *2* 2,3-dihydroxypropyl hexadecenoate; *3* 2,3-dihydroxypropyl palmitate; *4* 2,3-dihydroxypropyl margarate; *5* 2,3-dihydroxypropyl octadecenoate; *6* 2,3-dihydroxypropyl stearate; *7* 1-*O*-eicosyl-*sn*-glycerol; *8* 2,3-dihydroxypropyl eicosatetraenoate; *9* 2,3-dihydroxypropyl eicosatrienoate; *10* 2,3-dihydroxypropyl eicosadienoate; *11* 1-*O*-heneicosyl-*sn*-glycerol; *12*, *13* 1-*O*- docosyl-2,3-glycerol; *14*, *15*. 1-*O*-tricosyl-*sn*-glycerol

To confirm that alkylglycerols identified on the basis of GC–MS structural analysis (Fig. 3a) belong to ether-type lipids, they were subjected to alkaline methanolysis and acid methanolysis. In both conditions of the degradation procedures, some HF-released lipids were insensitive to solvolysis (Fig. 3b, c). Based on these results, it can be deduced that they do not belong to the sphingosine-, plasmalogen-, or acylglycerol-type of lipids because they are not stable under such treatment [17]. The only class of lipids exhibiting stability under such hydrolytic conditions is glycerol ethers in which alkyl side chains are linked to the glycerol backbone through stable ether linkages. The peaks marked as 7, 11, 12, 13, 14, and 15 corresponding to alkylgycerols, presented in Table 1, were observed in all ionograms. However, some peaks (1, 3, 6) corresponding to TMS ethers of saturated monoacylglycerols (2,3-dihydroxypropyl myristate, 2,3-dixydroxypropyl palmitate, 2,3-dixydroxypropyl stearate) were still identified after alkaline methanolysis (Fig. 3c). The monoglyceride content among the alkaline solvolysis products was probably caused by equilibrium reactions of transesterification [32–34], since peaks corresponding exclusively to monoalkylglycerols were observed after acid methanolysis (Fig. 3b). Besides, peaks corresponding to methyl esters of fatty acids (saturated C14–19:0, and unsaturated 16:1, 18:1, 20:2, 20:3, 20:4, 30:2, 30:3) appeared in the chromatogram of methanolysis products. This kind of fatty acids were identified previously [35] in the acylglycerol-type of lipids in *Acanthamoeba castellanii*, which might confirm that they originated from monoacylglycerols in our sample. This result was well correlated with disappearance of all peaks corresponding to monacylglycerols (both 1- and 2-isomers).

### Ether Cleavage

**Fig. 4:**
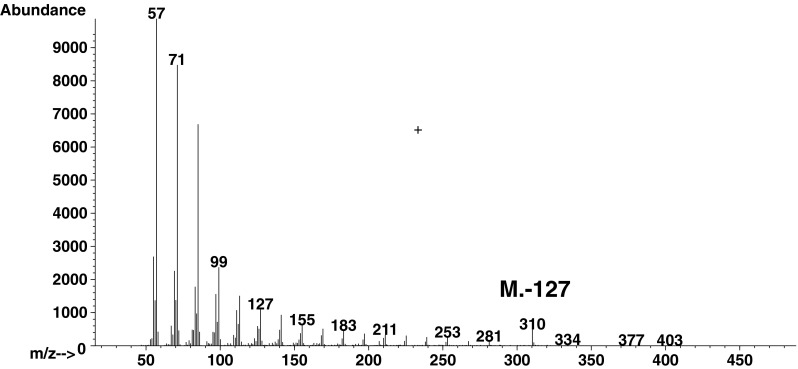
The mass spectrum of docosyl iodide derived from C_22_-AKG after hydrolysis with HI. [M-127] represents the ion with loss of iodide

The presence of ether-bound alkyl side chains in the HF-liberated lipids (spot D) was confirmed by cleavage thereof with hydroiodic acid [17]. The resulting alkyl iodides were directly analyzed by GC–MS. Although it is known that GC–MS analysis operating in the EI mode shows limits of detection of this kind of compound [36] by the lack of molecular ions, the loss of iodide [M + H − I]^+^ as the ions at 282, 296, 310, 324 *m*/*z* for alkyl chains with particular lengths were observed in the spectra of the derivative products (Fig. 4). This kind of fragmentation process is the most important for the heavier alkyl halides (including iodine) and involves simply losing the halogen to form an alkyl carbocation (*ipso*-cleavage) [37]. According to the spectra of alkyl iodides, eicosyl-, heneicosyl-, docosyl- and tricosyl iodides, respectively, were identified.

### GC–MS Analysis of Periodate-treated Lipids

**Fig. 5:**
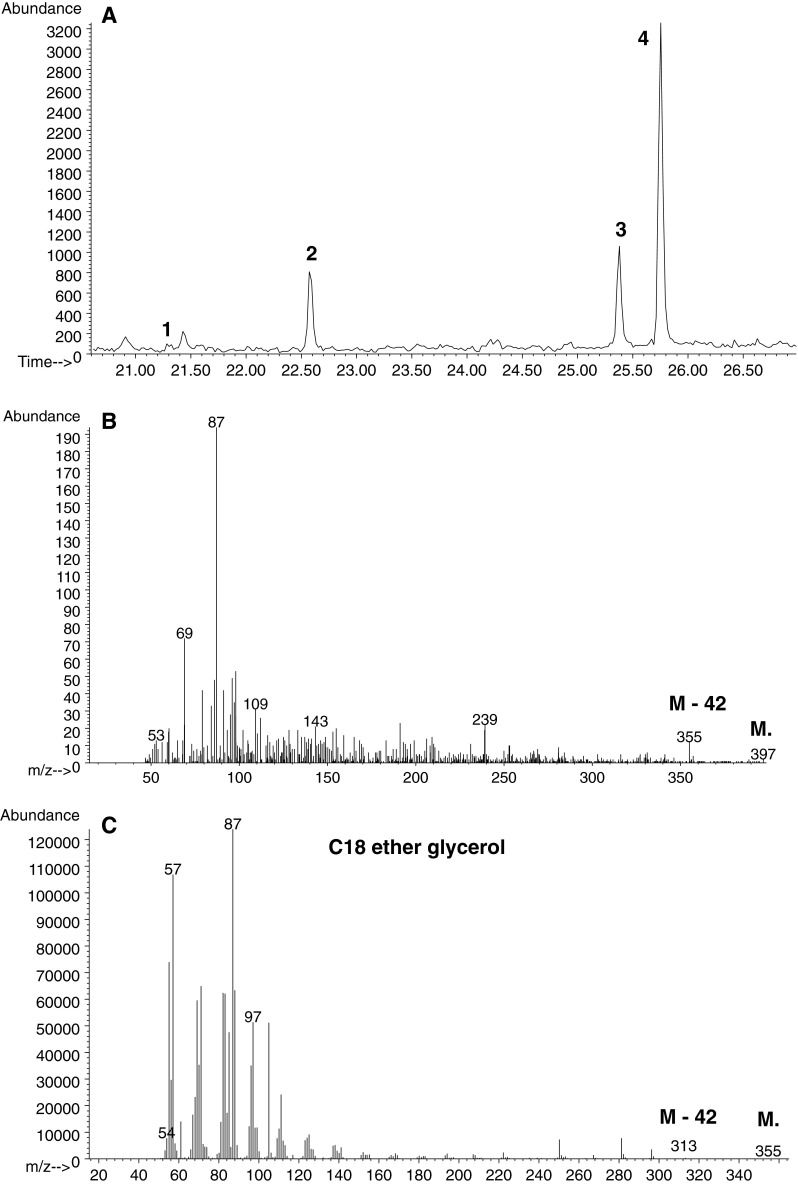
Ionogram for the ion at *m*/*z* 87 of 2-acetyl glycols obtained after periodate treatment, reduction and acetylation of ether glycerol lipids HF liberated from *A. rhysodes* cells (**a**) and mass spectra of glycols derived from (**b**) 1-*O*-heneicosyl-*sn*-glycerol (peak 2), **c** authentic standard. The number of *marked peaks* denotes glycols obtained from *1* 1-*O*-eicosyl-*sn* -glycerol, *2* 1-*O*-heneicosyl-*sn*-glycerol, *3, 4* 1-*O*-docosyl-*sn*-glycerol

The HF treatment of whole *A. rhysodes* cells liberated seven classes of monoalkylglycerols (Table 1). To determine definitively whether the alkyl substituents in ether lipids were at the C2 or C3 position of the glycerol backbone, lipids from spot D were treated with periodate, followed by reduction and acetylation to obtain appropriate radyl-glycol acetates. The resulting products were analyzed by GC–MS. The chromatography profile analysis of glycols (Fig. 5a) and TMS derivatives of AKG (Fig. 3b) showed close similarities, except for the retention times for the individual particles changed by the mass reduction in the case of glycerol residue subjected to oxidative cleavage. No molecular ions of radyl-glycols were formed in the EI mode, but ions with loss of ketene [M-42]^+^ were identified for almost all glycols derived from normal and branched 1-*O*-alkylglycerols treated with periodate (Fig. 5b). Exceptionally, no peak for normal and branched glycols derived from tricosanyl glycerols were observed in the chromatogram of ether lipids. This may have been caused by the small amount of the sample, higher masses of the derivative products, and poor efficiency of oxidation. Since all the monoalkylglycerols showed similar spectra as the TMS ethers with the characteristics of the 1,2-diol ion at *m*/*z* 205, it can be concluded that tricosanyl glycerol had a side chain in the *sn*-1 position as well. Therefore, the spectra obtained confirmed that all the alkyl moieties were substituted at the *sn*-1 position of the glycerol backbone.

### Isolation of Glycoinositolphospholipids (GIPL) from Amoeba Membrane

The crude membrane obtained according to the method of Korn et al. [11] was subjected to extraction with hot phenol. The material from the aqueous phase [15] was delipidated with a chloroform–methanol solvent and washed with acetone [11]. All the procedures were applied to remove proteins and neutral and polar lipids from *A. rhysodes* plasma membrane. Subsequently, extraction with 9 % *n*-butanol was applied to obtain glycoinositolphospholipids and partition with the *n*-butanol-water mixture to separate LPG (water fraction) from residual free GPI (butanol fraction) [16] (Scheme 1). When analyzed by SDS polyacrylamide gel electrophoresis, the LPG obtained during the purification steps yielded two silver stained bands of high electrophoretic mobility, typical for this polymer. The chromatographic analysis of the fatty acids released by acid hydrolysis from LPG showed exclusively long chain saturated normal and branched FA (C_16–28_) and normal and branched α-hydroxy (C_20–28_) characteristic for LPG of *Acanthamoeba* [14].

### Identification of Ether Lipids in a Backbone of Glycoinositolphospholipids

**Fig. 6:**
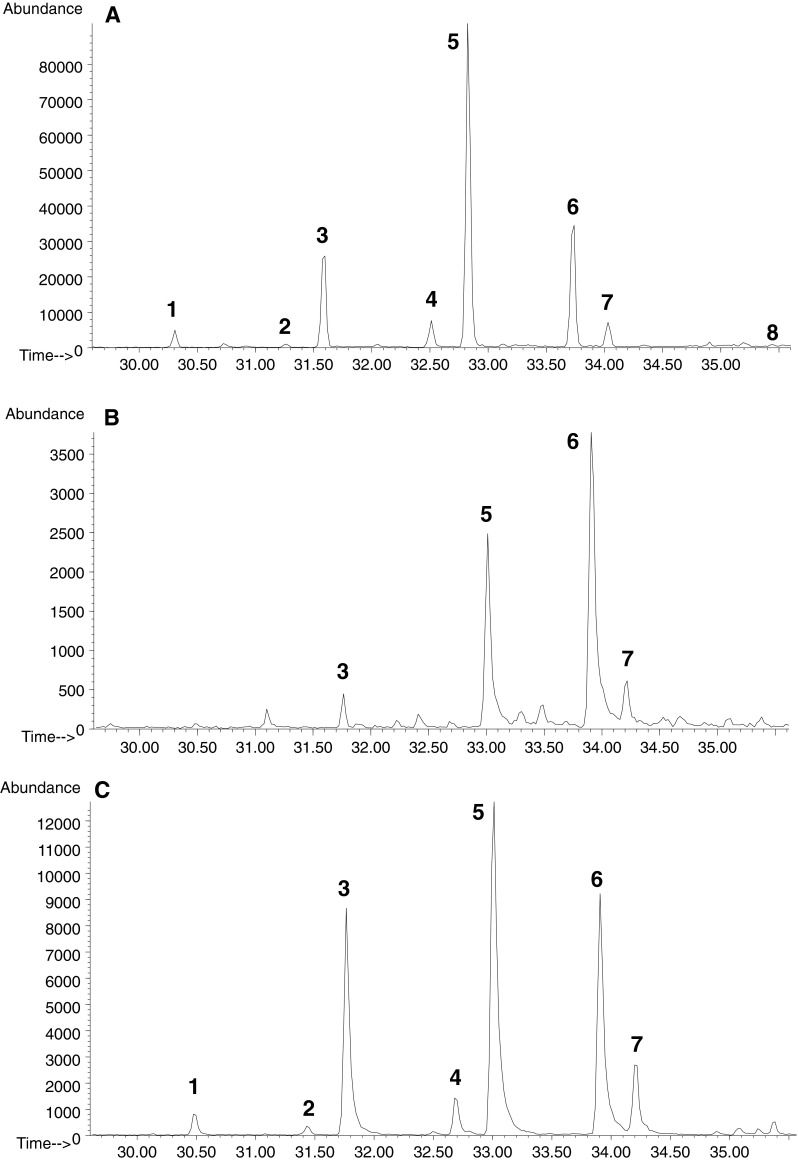
Ionograms for the ion at *m*/*z* 205 of TMS ethers of monoalkylglycerols liberated in acid methanolysis according to Watanabe et al. [21]. **a** From GIPL, **b** LPG, **c** GPI of *A. rhysodes*. The number of *marked peaks* denotes TMS derivatives of ether lipids: *1* C_20_; *2*, *3* C_21_; *4*, *5* C_22_; *6*, *7* C_23_; *8* C_24_

Samples of GIPL, LPG, and GPI were subjected to methanolysis according to Watanabe et al. [21], analyzed by GC–MS, and examined for ether lipids as TMS ether derivatives. Among the products released from GIPL, eight peaks corresponding to C_20–24_ ether lipids were recorded (Fig. 6a). The hydrolysis of LPG liberated only four products related to 1-monoalkylglycerols with the alkyl side chain C_21–23_, and the most abundant was the only branched AKG—1-*O*-docosyl-*sn*-glycerol in the pool (Fig. 6b). The other three AKGs had attached normal hydrocarbons. Chromatographic analysis of free GPI subjected to hydrolysis revealed seven species of AKG with a profile almost identical to that obtained from GIPL (Fig. 6c).

**Fig. 7:**
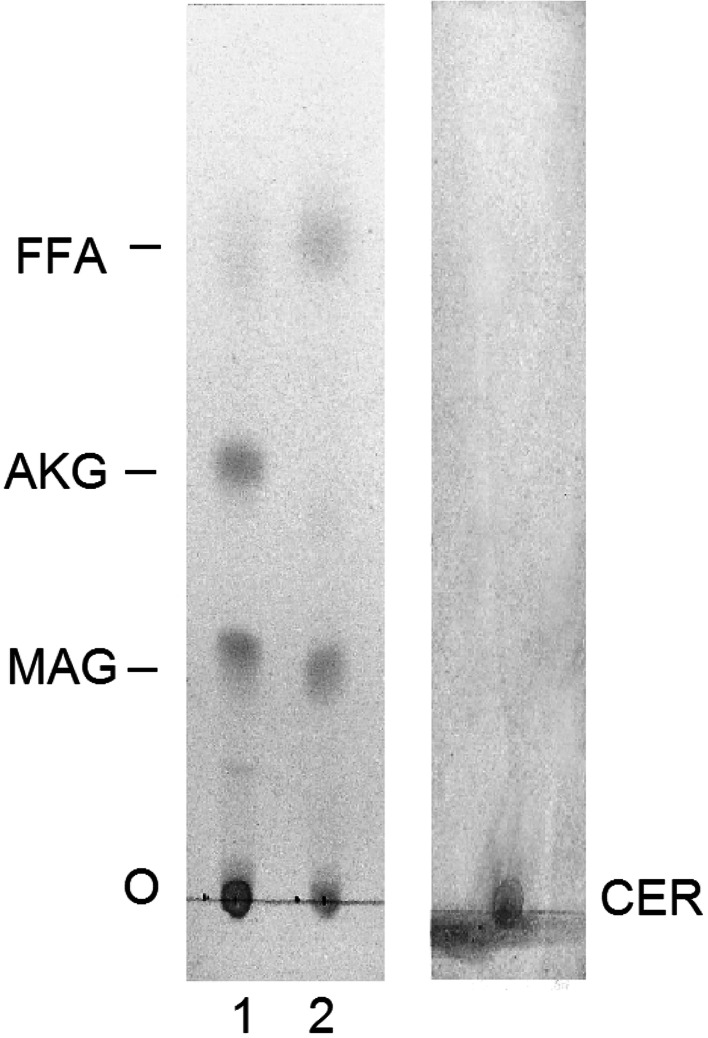
TLC analysis of lipids liberated from GIPL: *left panel.* *Line 1* With HF; *line 2* After methanolysis according to Watanabe et al. [21]. Lipids separated as isopropylidene derivatives in solvent 2 and visualized with iodine vapour. *Right panel.* Isopropylidene derivative of authentic standard phytoceramide—*t*18:0(24:0) visualized with sulfuric acid. *FFA* Free fatty acids, *AKG* monoalkylglycerols, *MAG* monoacylglycerols, *CER* phytoceramides

The GIPL sample was exposed to dephosphorylation with HF to liberate the lipid part. Subsequently, exhaustive extraction of the liberated products was carried out with different solvents. Lipid moieties obtained from GIPL were converted to isopropylidene derivatives and separated in TLC using solvent system B. Two spots were observed on the plate with *R*_F_ 0.59 and 0.27 corresponding to fatty acids and MAG, respectively. Additionally, no spot with *R*_F_ 0.42 corresponding to monoalkylglycerols was revealed (Fig. 7). The spot for AKG, except for FA and MAG, appeared when the products liberated by HF from GIPL were subjected to acid hydrolysis prior to conversion to isopropylidene derivatives and TLC (Fig. 7). The same results were obtained when lipids liberated from GIPL through dephosphorylation were silylated and directly analyzed in GC–MS. No peak related to the TMS derivative of AKG was observed in the chromatogram. Besides, peaks corresponding to AKG were recorded when the same material was hydrolyzed and treated with TMS. This may suggest that ether lipids in the lipid part of GIPL are alkylacylglycerols, but not the *lyso*-alkyl type.

To confirm that the AKG identified are the lipid part of LPG, the sample of LPG was subjected to nitric acid deamination. Among the products liberated during the process, four classes of C_21–23_ AKG with a chromatographic profile similar to that obtained after acid methanolysis (Fig. 6b) and phytosphingosines *t*25:0–27:0 (normal and branched) reported earlier as being a part of the ceramide moiety of LPG were identified [14]. Additionally, butanol soluble products liberated through deamination contained inositol, whereas 2,5-anhydromannitol derived from unsubstituted glucosamine was identified among the water-soluble ones [38]. These results show that the ether glycerol lipid moiety is bound with GlcN through phosphoinositol, which proves that it is a part of the LPG polymer.

## Discussion

Glycerol based ether lipids are normally minor constituents of most cell membranes in mammals; in contrast, they are major constituents in cell membranes of archaean [39] and some protozoan parasites [16]. The most prevalent ether backbones found in nature consist of 1-*O*-alkyl or 1-*O*-alk-1′-enyl. Until recently, they have been considered to be little more than a biological novelty. However, findings of elevated levels of ether lipids in cancer tissues, followed by the discovery of distinctive ether lipids, such as platelet-activating factor (PAF), with important biological activities have greatly stimulated the interest in these compounds. Both synthetic and naturally occurring alkylglycerols themself possess several potent pharmacological activities. In vitroand in vivo studies of ether lipids have shown that they mediate immune response. 1-*O*-Dodecylglycerol in trace amounts (0.05 μg/mL) was able to induce markedly elevated ingestion activity of macrophages. In turn, higher amounts thereof were toxic. It is a potent gram-positive antibacterial agent effectively acting at a relatively low concentration (4 μg/mL) against *Streptococcus faecium* ATCC 9790 through stimulation of peptidoglycan hydrolase (autolysin) as well [1]. A synergistic effect of 1-*O*-dodecylglycerol with penicillin G was shown in experiments with the same and other species of bacteria [40]. A similar event was observed with amphotericin B (antifungal agents) [41]. As a part of diacyl glyceryl ethers, AKG was also shown to strongly inhibit the growth of three prostate cancer cells (Du-145, PC-3, PCa-2b) [1]. On the other hand, ether lipid levels in other types of cancer cells (e.g. breast cancer) are elevated. Their specific function remains unclear but inactivation of the alkylglyceronephosphate synthase (a key enzyme in the biosynthesis of glycerol-ether lipids) led to significant impairments in cancer pathogenicity and its overexpression increased cancer cell motility, survival, and tumor growth [42]. Ether lipids are highly abundant in nervous tissues and play a major role as cellular antioxidants. Changed levels are associated with neurological dysfunctions and degeneration [43]. Other functions of ether lipids are not well understood, but they have been implicated in maintaining physiochemical properties of cell membranes, such as fluidity, fusion events, and lipid raft domains. More examples of the action of glyceryl-ether lipids (such as reduction of injuries, increasing cell permeability, BBB opening) can be found in the very comprehensive review by Iannitti and Palmieri [44].

Ether linked hydrocarbons in parasitic protozoa membranes can occur as constituents of phospholipids and GPI anchored glycoconjugates and glycoproteins. As such, they are involved in many aspects of host-parasite interactions, such as adhesion and invasion of host cells, modulation and evasion from host immune response, and pathogenesis [16].

Cosmopolitan amoebae belonging to the genus *Acanthamoeba* are the causative agents of some diseases (AK, GAE). Some strains, both virulent and avirulent, can also come into close interactions with pathogenic bacteria, viruses, yeasts, or protists. It is known that amoeba cells are covered with a GPI-anchored polysaccharide called LPG. In the past, 77 % of its constituents were identified but the rest stayed unexplored [12]. It was also established that LPG has a polymorphic nature since two types of LPG differing in the length and composition of oligosaccharide attached to the lipid portion were identified. The lipid part of LPG was established to be a ceramide type with phytosphingosine moieties *t*24–28:0 substituted with normal and 2-hydroxy long chain fatty acids [12, 14]. Current results presented in this report have demonstrated that ether-type alkylacylglycerol moieties exist in LPG as well. They were established to be C_21–23_ in length and the most abundant were those substituted with the *anteiso*-C_23_ (57.26 %) and nC_22_ (30.12 %) alkyl chain. Since electrophoretic analysis of LPG still showed only two bands, with migration presented earlier as typical for that kind of a polymer [12], it seems very plausible that different types of lipid moieties are attached to the same oligosaccharide parts. Lederkremer et al. [45] demonstrated that the GIPL lipid domain structure, in epimastigotes of *T. cruzi* strain CL collected at the exponential phase of growth, is developmentally regulated, and alkylacylglycerol (1-*O*-hexadecyl-2-*O*-palmitoylglycerol) or ceramide (sphinganine-containing) were found linked to the same glycan. In turn, *Saccharomyces cerevisiae* glycoproteins are anchored by either a glycerolipid or a C_18_-phytosphingosine-containing ceramide. A suggestion has been made that the base sensitive lipid moiety is exchanged by ceramide during glycoprotein maturation [46].

The chromatographic profile of AKG derived from free GPI of *A. rhysodes* showed seven peaks identified as monoalkylglycerols with attached hydrocarbons with a length of C_20–24_ (Fig. 6c) and the predominant was that with an attached C_22_ hydrocarbon (47.56 %). They were also identified as AAG moieties. The distribution of particular ether lipids for LPG and GPI was distinct. In LPG preparations, the most abundant AKG were those with the longest branched hydrocarbon chain (C_23_), while in GPI those with a shorter normal one (C_22_). Similar results were obtained for *Leishmania major* glycolipids which generally contained C_18_, C_22_, C_24_, and C_26_ alkyl chains, although there was an increase in the proportion of C_24_ and C_26_ hydrocarbons with elongation of the carbohydrate chain [3]. In *Acanthamoeba*, the LPG polymer carries longer than GPI oligosaccharide moieties; therefore, the same rule as in *Leishmania* may apply. GPI moieties in protozoa cells can exist as free structures in the membrane or serve as protein anchors [45, 47]. An important feature of the free GPI/GIPL structures developed by *Leishmania* and *Trypanosoma cruzi*, in contrast to those of *Plasmodium falciparum* and *Trypanosoma brucei*, is the preponderance of alkylacylglycerols (or *lyso*-alkylglycerols) and ceramides, as opposed to diacylglycerols, within the lipid domain [47]. The differences in the chemical structure have their implications in the possible function. In preliminary studies, it was established that GPI/GIPL containing a ceramide or a saturated lipid chain in the alkylacyl-PtdIns and *lyso*-alkyl-PtdIns moieties severely inhibit macrophages, while that derived from *P. falciparum* and *T. brucei* are potent macrophage activating molecules [4, 5, 47, 48]. The mechanism of mediating immune responses can rely on inhibition of PKC action through ether lipids or ceramide moieties in contrast to the lack of an effect served by dialkyl glycerols [5, 49, 50]. Thus, LPG of *Acanthamoeba* containing both the ceramide and alkylacylglycerol type of the lipid portion may be able to subvert the immune system and control the infection of the brain.

When whole cells of *A. rhysodes* were subjected to dephosphorylation with HF, seven classes of *lyso*-alkylglycerols (C_20–23_) were released. Since, parasitic protozoa possess, among others, ether-linked hydrocarbons as components of phospholipids [51], and because during our investigations ether lipids were liberated from GIPL of *A. rhysodes* established exclusively as the alkylacylglycerol type, we deduced that monoalkylglycerols originated from the phospholipid fraction. Hydrolizates of the phospholipid fraction (data not presented) showed a similar chromatographic profile as the dephosphorylated material. However, exact determination of ether phospholipids will be the subject of our future studies. It is also known that parasitic protozoa secrete exosomes, which are proposed to act as messengers to prime host-cells preparing the host for the incoming parasite. Vesicles may arise from plasma membrane budding, which liberates membrane fragments that ultimately form vesicles by fusion of their extremities [52]. The mammalian brain enzymes selective for the plasmanyl and plasmenyl type of phospholipids can be engaged in that process because they participate in creating another type of membrane vesicle—synaptosomes [53]. Taken together with the findings that microvesicles are shed by *T. cruzi* and engulfed by the host cells to prepare them for the incoming trypanosome, it suggests a potential role of the phospholipid-containing ether moiety from *Acanthamoeba* in preparing endothelial cells in the BBB to traversing by amoebae. These all are only hypothetical functions of ether lipids that need to be confirmed in future studies.

## Abbreviations

AEP: 2-Aminoethylphosphonate
AK: Acanthamoeba keratitis
AAG: Alkylacylglycerol
AKG: Alkylglycerol
BBB: Blood-brain barrier
CER: Phytoceramide
DHB: 2,5-Dihydrobenzoic acid
EI: Electron impact
FFA: Free fatty acid(s)
FAME: Fatty acid methyl ester(s)
GAE: Granulomatous amoebic encephalitis
GC–MS: Gas chromatography–mass spectrometry
GIPL: Glycoinositolphospholipid
GPI: Glycosylphosphatidylinositol anchor
HF: Hydrofluoric acid
LPG: Lipophosphonoglycan
MAG: Monoacylglycerol
MALDI-TOF: Matrix-assisted laser desorption/ionization time-of-flight
MeOH: Methanol
PAF: Platelet-activating factor
PKC: Protein kinase C
PtdIns: Phosphatidylinositol
PYG: Peptone yeast glucose medium
SDS: Sodium dodecyl sulfate
TLC: Thin layer chromatography
TMS: Trimethylsilyl group

## References

[1] Magnusson CD, Haraldsson GG (2011). Ether lipids. Chem Phys Lipids.

[2] McConville MJ, Bacic A (1989). A family of glycoinositol phospholipids from *Leishmania major*. J Biol Chem.

[3] McConville MJ, Homans SW, Thomas-Oates JE, Dell A, Bacic A (1990). Structures of glycoinositolphospholipids from *Leishmania major*. A family of novel galactofuranose-containing glycolipids. J Biol Chem.

[4] Chawla M, Vishwakarma RA (2003). Alkylacylglycerolipid domain of GPI molecules of *Leishmania* is responsible for inhibition of PKC-mediated c-*fos* expression. J Lipid Res.

[5] McNeely TB, Rosen G, Londner MV, Turco SJ (1989). Inhibitory effect on protein kinase C activity by lipophosphonoglycan fragments and glycosylphosphatidylinositol antigens of the protozoan parasite *Leishmania*. Biochem J.

[6] Erdlenbruch B, Alipour M, Fricke G, Miller DS, Kugler W, Eibl H, Lakomek M (2003). Alkylglycerol opening of the blood–brain barrier to small and large fluorescence markers in normal and C6 glioma-bearing rats and isolated rat brain capillaries. Br J Pharmacol.

[7] Erdlenbruch B, Schinkhof C, Kugler W, Heinemann DEH, Herms J, Eibl H, Lakomek M (2003). Intracarotid administration of short-chain alkylglycerols for increased delivery of methotrexate to the rat brain. J Pharmacol.

[8] Hooper NM (2004). Encyclopedia of genetics, genomics, proteomics and bioinformatics. Part 3. proteomics. 3.5. proteome diversity. Wiley Online Libr.

[9] Guha-Niygoi A, Sullivan DR, Turco SJ (2001). Glycoconjugate structures of parasitic protozoa. Glycobiology.

[10] Ulsamer AG, Smith FR, Korn ED (1969). Lipids of *Acanthamoeba castellanii*. Composition and effects of phagocytosis on incorporation of radioactive precursors. J Cell Biol.

[11] Korn ED, Dearborn DG, Wright PL (1974). Lipophosphonoglycan of the plasma membrane of *Acanthamoeba castellanii*. J Biol Chem.

[12] Dearborn DG, Smith S, Korn ED (1976). Lipophosphonoglycan of the plasma membrane of *Acanthamoeba castellanii*. J Biol Chem.

[13] Band RN (1959). Nutritional and related biological studies on the free-living soil amoeba, *Hartmannella rhysodes*. J Gen Microbiol.

[14] Karaś M, Russa R (2013). New long chain bases in lipophosphonoglycan of *Acanthamoeba castellanii*. Lipids.

[15] Previato JO, Gorin PAJ, Mazurek M, Xavier MT, Fournet B, Wieruszesk JM, Mendonҫa-Previato L (1990). Primary structure of the oligosaccharide chain of lipopeptidophosphoglycan of epimastigote forms of *Trypanosoma cruzi*. J Biol Chem.

[16] Nakayasu ES, Yashunsky DV, Nohara LL, Torrecilhas ACT, Nikolaev AV, Almeida IC (2009). GPIomics: global analysis of glycosylphosphatidylinositol-anchored molecules of *Trypanosoma cruzi*. Mol Syst Biol.

[17] Orlandi PA, Turco SJ (1987). Structure of the lipid moiety of the *Leishmania donovani* lipophosphoglycan. J Biol Chem.

[18] Wood R (1967). GLC and TLC analysis of isopropylidene derivatives of isomeric polyhydroxy acids derived from positional and geometrical isomers of unsaturated fatty acids. Lipids.

[19] Warne TR, Buchanan FG, Robinson M (1995) Growth-dependent accumulation of monoalkylglycerol in Madin-Darby canine kidney cells. Evidence for a role in the regulation of protein kinase C. J Biol Chem 270 (19):11147-11154 http://www.jbc.org/content/270/19/11147 10.1074/jbc.270.19.11147 7744745

[20] Rütters H, Sass H, Cypionka H, Rullkötter J (2001). Monoalkylether phospholipids in the sulfate-reducing bacteria *Desulfosarcina variabilis* and *Desulforhabdus amnigenus*. Arch Microbiol.

[21] Watanabe Y, Nakajima M, Hoshino T, Jayasimhulu K, Brooks EE, Kaneshiro ES (2001). A novel sphingophosphonolipid head group 1-hydroxy-2-aminoethyl phosphonate in *Bdellovibrio stolpii*. Lipids.

[22] Caroff M, Chaby R, Karibian D, Perry J, Deprun C, Szabo L (1990). Variations in the carbohydrate regions of *Bordetella**pertussis* lipopolysaccharides: electrophoretic, serological and structural features. J Bacteriol.

[23] Lesse AJ, Campagnari AA, Bittner WE, Apicella MAJ (1990). Increased resolution of lipopolysaccharides and lipooligosaccharides utilizing Tricine-sodium dodecyl sulfate-polyacrylamide gel electrophoresis. J Immunol Methods.

[24] Tsai CM, Frasch CE (1982). A sensitive silver stain for detecting lipopolysaccharides in polyacrylamide gels. Anal Biochem.

[25] Zenker A, Pfanzagl B, Löffelhardt W, Allmaier G (1998). Negative and positive ion matrix-assisted laser desorption ionization mass spectrometry of peptidoglycan fragments after size fractionation and reversed-phase high-performance liquid chromatography. J Microbiol Methods.

[26] Saito R, Oba M, Kaiho K, Maruo Ch, Fujibayashi M, Chen J, Chen ZQ, Tong J (2013). Ether lipids from the Lower and Middle Triassic at Qingyan, Guizhou Province, Southern China. Org Geochem.

[27] Bertello LE, Gonҫalvez MF, Colli W, de Lederkremer RM (1995). Structural analysis of inositol phospholipids from *Trypanosoma cruzi* epimastigote forms. Biochem J.

[28] Myher JJ, Marai L, Kuksis A (1974). Identification of monoacyl- and monoalkylglycerols by gas-liquid chromatography-mass spectrometry using polar siloxane liquid phases. J Lipid Res.

[29] Coelho D, Marques G, Gutiérrez A, Silvestre AJD, del Río JC (2007). Chemical characterization of the lipophilic fraction of giant reed (*Arundo donax*) fibres used for pulp and paper manufacturing. Ind Crop Prod.

[30] Johnson CB, Holman RT (1966). Mass spectrometry of lipids. II. Monoglycerides, their diacetyl derivatives and their trimethylsilyl ethers. Lipids.

[31] Ratnayake WMN, Timmins A, Ohshima T, Ackman RG (1986). Mass spectra of fatty acid derivatives, of isopropylidenes of novel glyceryl ethers of cod muscle and of phenolic acetates obtained with the Finnigat MAT Ion Trap Detector. Lipids.

[32] Barakos N, Pasias S, Papayannakos N (2008). Transesterification of triglycerides in high and low quality oil feeds over an HT2 hydrotalcite catalyst. Bioresour Technol.

[33] Limpanuparba T, Punyainb K, Tantirungrotecha Y (2010). A DFT investigation of methanolysis and hydrolysis of triacetin. J Mol Struct THEOCHEM.

[34] Asakuma Y, Kawanami O, Maeda K, Kuramochi H, Fukui K (2011). Theoretical study of the transesterification of triglycerides to biodiesel fuel under various conditions. IJOT.

[35] Palusińska-Szysz M, Turska-Szewczuk A, Karaś M, Russa R, Drożański WJ (2009). Occurrence of new polyenoic very long chain acyl residues in lipids from *Acanthamoeba castellanii*. Acta Protozool.

[36] Leclercq PA, Snijders HMJ, Crarners CA, Maurer KH, Rapp U (1989). Rapid and Ultra-Sensitive GC/MS Analyses with a Microchannel Plate Array Detector. Part I: possibilities of Simultaneous Ion Detection in Narrow-Bore GC/MS. J High Resolut Chrom.

[37] Central Connecticut State University. Department of Chemistry and Biochemistry. Mass Spectrometry—halogenated compounds. http://www.chemistry.ccsu.edu/glagovich/teaching/316/ms/halogen.html. Accessed 1 Mar 2013

[38] Karaś M, Russa R (2009). Localization of the attachment site of oligoglucans to *Mesorhizobium loti* HAMBI 1148 murein. Acta Biochim Pol.

[39] Koga Y, Mori H (2005). Recent advances in structural research on ether lipids from *Archaea* including comparative and physiological aspects. Biosci Biotechnol Biochem.

[40] Ved HS, Gustow E, Mahadevans V, Pieringer RA (1984). Dodecylglycerol. A new type of antibacterial agent which stimulates autolysin activity in *Streptococcus faecium* ATCC 9790. J Biol Chem.

[41] Haynes MP, Buckley HR, Higgins ML, Pieringer RA (1994). Synergism between the antifungal agents amphotericin B and alkyl glycerol ethers. Antimicrob Agents Chemother.

[42] Benjamina DI, Cozzoa A, Jib X, Robertsa LS, Louiea SM, Mulvihilla MM, Luob K, Nomuraa DK (2013). Ether lipid generating enzyme AGPS alters the balance of structural and signaling lipids to fuel cancer pathogenicity. PNAS.

[43] Engelmann B (2004). Plasmalogens: targets for oxidants and major lipophilic antioxidants. Biochem Soc Trans.

[44] Iannitti T, Palmieri B (2010). An update on the therapeutic role of alkylglycerols. Mar Drugs.

[45] Lederkremer RM, Lima CE, Ramirez MI, Gonçalvez MF, Colli W (1993). Hexadecylpalmitoylglycerol or ceramide is linked to similar glycophosphoinositol anchor-like structures in *Trypanosoma cruzi*. Eur J Biochem.

[46] Sipos G, Puoti A, Conzelmann A (1994). Glycosylphosphatidylinositol membrane anchors in *Saccharomyces cerevisiae*: absence of ceramides from complete precursor glycolipids. EMBO J.

[47] Tachado SD, Mazhari-Tabrizi R, Schofield L (1999). Specificity in signal transduction among glycosylphosphatidylinositols of *Plasmodium falciparum*, *Trypanosoma brucei*, *Trypanosoma cruzi* and *Leishmania* spp. Parasite Immunol.

[48] Ropert C, Gazzinelli RT (2000). Signaling of immune system cells by glycosylphosphatidylinositol (GPI) anchor and related structures derived from parasitic protozoa. Curr Opin Microbiol.

[49] Castrillo A, Pennington DJ, Otto F, Parker PJ, Owen MJ, Bosca L. 2001. Protein Kinase C is required for macrophage activation and defense against bacterial infection. J Exp Med 194:1231-1242 http://www.jem.org/cgi/content/full/194/9/1231 10.1084/jem.194.9.1231 PMC2195971 11696589

[50] Bourbon NA, Yun J, Berkey D, Wang Y, Kester M (2001). Inhibitory actions of ceramide upon PKC-epsilon/ERK interactions. Am J Physiol Cell Physiol.

[51] Kaneshiro ES, Guo Z, Sul D, Kallam KA, Jayasimhulu K, Beach DH (1998). Characterization of *Pneumocystis carinii* and rat lung lipids: glyceryl ethers and fatty alcohols. J Lipid Res.

[52] Torrecilhas AC, Schumacher RI, Alves MJ, Colli W (2012). Vesicles as carriers of virulence factors in parasitic protozoan diseases. Microbes Infect.

[53] Rosenberger TA, Oki J, Purdon AD, Rapoport SI, Murphy EJ (2002). Rapid synthesis and turnover of brain microsomal ether phospholipids in the adult rat. J Lipid Res.

